# Prevalence and factors associated with cracked nipples in the first month postpartum

**DOI:** 10.1186/s12884-016-0999-4

**Published:** 2016-08-05

**Authors:** Kamila Juliana da Silva Santos, Géssica Silva Santana, Tatiana de Oliveira Vieira, Carlos Antônio de Souza Teles Santos, Elsa Regina Justo Giugliani, Graciete Oliveira Vieira

**Affiliations:** 1 State University of Feira de Santana, Bahia, Brazil; 2 Federal University of Rio Grande do Sul, Rio Grande do Sul, Brazil; 3 Centro de Pós-Graduação em Saúde Coletiva, Mestrado Acadêmico, Avenida Transnordestina, s/n, Avenida dos Laboratórios, Módulo 6, Bairro Novo Horizonte, CEP 44036-900 Feira de Santana, BA Brazil

**Keywords:** Breastfeeding, Lactation disorders, Nipple injuries

## Abstract

**Background:**

To assess the prevalence and factors associated with the occurrence of cracked nipples in the first month postpartum.

**Methods:**

This was a cross-sectional study nested in a cohort of mothers living in Feira de Santana, state of Bahia, northeastern Brazil. Data from 1,243 mother-child dyads assessed both at the maternity ward and 30 days after delivery were analyzed. The association between cracked nipples as reported by mothers and their possible determinants was analyzed using Poisson regression in a model where the variables were hierarchically organized into four levels: distal (individual characteristics), distal intermediate (prenatal characteristics), proximal intermediate (delivery characteristics), and proximal (postnatal characteristics).

**Results:**

The prevalence of cracked nipples was 32 % (95 % confidence interval [95 % CI] 29.4–34.7) in the first 30 days postpartum. The following factors showed significant association with the outcome: poor breastfeeding technique (prevalence ratio [PR] = 3.18, 95 % CI 2.72–3.72); breast engorgement (PR = 1.70, 95 % CI 1.46–1.99); birth in a maternity ward not accredited by the Baby-Friendly Hospital Initiative (PR = 1.51, 95 % CI 1.15–1.99); cesarean section (PR = 1.33, 95 % CI 1.13–1.57); use of a feeding bottle (PR = 1.29, 95 % CI 1.06–1.55); and higher maternal education level (PR = 1.23, 95 % CI 1.04–1.47).

**Conclusions:**

The prevalence of cracked nipples was high in our sample. Most of the factors associated with cracked nipples were related to postnatal characteristics, especially poor breastfeeding technique, which could be improved to help prevent the condition.

## Background

Nipple trauma presents a high incidence, especially in the first 30 days postpartum [1, 2]. Because it is a painful condition, it often causes interruption of exclusive breastfeeding and early weaning [3, 4]. In the city of New York, 35 % of the women stopped breastfeeding within a week after birth due to nipple trauma, and 30 % between 1 and 4 weeks postpartum [3]. Similarly, in the municipality of Feira de Santana, northeastern Brazil, a 25 % higher risk of interruption of exclusive breastfeeding was found in the first month of lactation when cracked nipples were present [4].

Several factors have been identified as determinants of nipple trauma in breastfeeding mothers, e.g., poor breastfeeding technique/position/latch-on [2, 5, 6], use of a feeding bottle [1, 7], breast engorgement [8, 9], primiparity [8, 10], semi-protruding and/or malformed nipples [8, 9], use of breast pumps [11], and depigmented nipples [8], among others. Knowledge of the factors involved in the genesis of this condition in specific populations is extremely important to help establish preventive measures, especially because of the little effectiveness associated with the treatment options available [12, 13].

Therefore, the objective of the present study was to investigate the prevalence and factors associated with cracked nipples in the first 30 days postpartum in a cohort of women living in Feira de Santana, state of Bahia, Brazil. The results of the current study may contribute to the design of measures that could help prevent cracked nipples.

## Methods

### Study design

This was a cross-sectional study nested in a cohort study conducted with a sample of mother-child dyads residing in the municipality of Feira de Santana, a large city located in the state of Bahia, northeastern Brazil, 108 km distant from Salvador, the state capital [14]. In 2012, Feira de Santana had a population of around 568,000 inhabitants. The city is an important economic hub, especially in the fields of commerce, industry, and cattle breeding.

### Sample size and data collection

Sample size for this study was calculated considering the number of live births estimated for year 2003 in Feira de Santana (10,177), an incidence of cracked nipples of 51 %, as previously reported [15], an error of 5 %, and a 95 % confidence interval (95 % CI). Sample size calculation resulted in a minimum of 371 mother-child dyads, but 1,309 dyads were interviewed.

Mother-child dyads were recruited at all maternity hospitals serving the municipality of Feira de Santana, at a total of 10 services (public and private), over a 12-moth period, from April 2004 to March 2005. Two maternity hospitals were drawn each time and visited daily, for 2 consecutive months, except for two hospitals conducting a large number of deliveries, which were selected and visited individually. Inclusion criteria were being a resident of Feira de Santana, absence of intercurrences during pregnancy or in the immediate postpartum period, absence of contraindications to breastfeeding, children without any perinatal complication.

All mothers were approached by previously trained health professionals working at a human milk bank, and all signed an informed consent form before undergoing any study procedures. Mothers were interviewed at the maternity ward, and follow-up interviews were conducted at the mothers’ homes at 1, 2, 3, 4, 5, and 6 moths postpartum.

A total of 1,360 mother-child dyads were considered eligible for the study. Of those, 1,344 were included in the cohort (10 mothers refused to participate, 4 were unable to inform an address, and 2 resided in areas considered to be unsafe for the research team). At the first follow-up visit, at 30 days, 35 dyads (2.6 %) were lost, resulting in a total of 1,309 dyads interviewed. The dataset was then filtered to exclude cases with missing information, resulting in a final sample of 1,243 observations for analysis. The results reported in the present study refer to the data collected at the maternity ward and at the 30-day follow-up interview.

A form was developed based on a questionnaire approved by the Center of Excellence and Motivation for Breastfeeding of the Human Milk Bank at Hospital Geral Clériston Andrade, combined with additional questions used by the Brazilian Ministry of Health in the annual assessment of hospitals accredited by the Baby-Friendly Hospital Initiative.

### Variables

For the scope of the present study, cracked nipples were defined based on the mother’s report of the presence of cracks in the nipples over the first month postpartum, i.e., any type of damage (cracks, ulcers) to the nipple-areolar region [9], associated or not with pain while breastfeeding. This definition did not include pain while breastfeeding alone, i.e., without the presence of cracks. Breast engorgement was defined based on the mother’s report of turgid, tense, painful, swollen breasts [16, 17]. Skin color, breast engorgement, and breastfeeding within the first hour after delivery were self-reported by the mothers. Type of nipple was assessed by the interviewer and classified into protruding (normal) or non-protruding (flat/inverted/pseudo-inverted).

Breastfeeding technique was assessed in all dyads, regardless of the mother’s report of cracked nipples. Breastfeeding technique was classified as proper when the baby’s body was facing the mother’s body; when the baby’s mouth was wide open; when the baby’s chin touched the breast; when the baby’s lips were turned outward; when the nipple presented an elongated, but still round shape after the feed; and when nipple pain was absent. The breastfeeding technique was considered poor or inadequate when any of these criteria were not met.

Previous experience with breastfeeding was considered to be present when the mother reported having breastfed another child. Hospitals were considered to be Baby-Friendly when accredited by the Brazilian Ministry of Health’s and United Nations Children's Fund’s Baby-Friendly Hospital Initiative for following the guidelines established in the ten steps to successful breastfeeding.

All these data were collected both at the maternity ward and at the mothers’ homes at 30 days postpartum.

### Statistical analysis

**Fig. 1:**
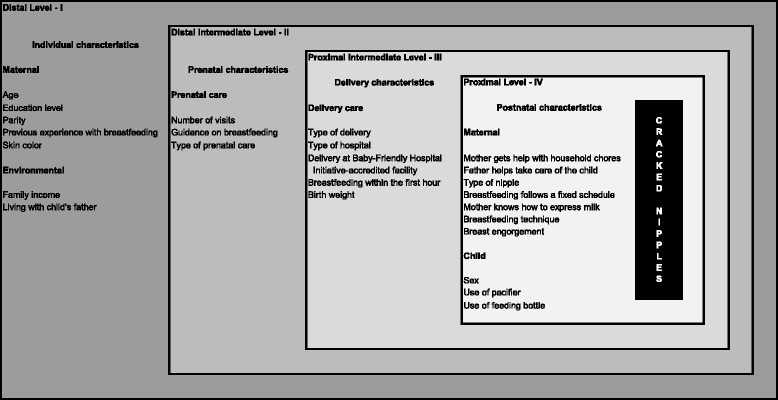
Hierarchical model of factors potentially associated with cracked nipples

In order to assess factors associated with cracked nipples, a conceptual model was established (Fig. 1) with factors hierarchically organized into four levels, according to their proximity to the dependent variable [18]. The variables comprising each level were chosen based on established scientific knowledge. In order not to exclude potential confounding factors, any variable reaching a level of significance ≤0.20 in any stage of the analysis was maintained in the model, even if losing statistical significance in subsequent stages. In the final model, only variables reaching a statistical significance of p ≤ 0.05 were considered to be associated with the outcome. Poisson regression analysis with robust variance estimation was used to estimate prevalence ratios (PR) and their respective 95 % CI.

In the first stage of regression analysis, the effect of variables included in the more distal level of the hierarchical model was analyzed, with no adjustment for variables belonging to the other levels of proximity. This more distal level was related to the life conditions of the sample assessed, i.e., maternal characteristics (age, education level, parity, previous experience with breastfeeding, skin color) and environmental characteristics (family income and living with the child’s father). The second stage of analysis included variables from the first stage that reached significance (p ≤ 0.20), plus variables belonging to the distal intermediate level of the hierarchical model, i.e., prenatal characteristics. A similar procedure was adopted in the third (delivery characteristics) and fourth (postnatal characteristics) stages. Therefore, the final model comprised all variables from the four hierarchical levels that showed a significant association (p ≤ 0.20) with the outcome of interest.

All data collected were double-entered into the Statistical Package for the Social Sciences (SPSS) version 10.0. Statistical analyses were performed in the STATA software version 8.0.

### Ethical aspects

The study was approved by the Research Ethics Committee of Universidade Estadual de Feira de Santana (protocol no. 012/2003). All women included in the study provided written informed consent. Mothers presenting any abnormalities on the clinical breast exam were referred for evaluation and follow-up at Clériston Andrade General Hospital.

## Results

### Prevalence of cracked nipples in the first month postpartum

The prevalence of cracked nipples was 32 % (398/1,243; 95 % CI 29.4-34.7), but all mothers were still breastfeeding at the 30-day follow-up interview, even in the presence of the outcome. Participation rate was 97.4 % (1,309/1,344), and another 66 were discarded due to dataset filtering to exclude cases with missing information, resulting in a final sample of 1,243 dyads included in the analysis.

### Factors associated with cracked nipples in the first month postpartum

According to Table 1, at the first stage of the hierarchical model, maternal education and family income remained in the model. At the second stage, type of prenatal care was associated with the outcome, and at the third stage, type of delivery and delivery at a Baby-Friendly Hospital Initiative-accredited facility. Breastfeeding technique, breast engorgement, and use of a feeding bottle at 30 days were the significant variables at the fourth stage of analysis. All these variables were included in the final model. Except for family income and type of prenatal care, all variables selected remained associated with the outcome, even after adjustment for other variables. Therefore, following completion of all analysis stages, the following variables resulted significantly associated with the presence of cracked nipples in the first month postpartum: higher maternal education level, cesarean section, birth at a Baby-Friendly Hospital Initiative non-accredited facility, poor breastfeeding technique, breast engorgement, and use of a feeding bottle.

**Table 1:** Prevalence of cracked nipples according to different variables in Poisson regression analysis

Variables	*N* (%)	Cracked nipples	First-stage	Second-stage	Third-stage	Fourth-stage	*p* ^a^
		*n* (%)	PR (95 % CI)	PR (95 % CI)	PR (95 % CI)	PR (95 % CI)	
Mother’s age							
10–19 years	235 (18.91)	79 (19.85)	1.00	-	-	-	
20–29 years	666 (53.58)	213 (53.52)	0.95 (0.76–1.18)	-	-	-	
30–39 years	321 (25.82)	100 (25.13)	0.99 (0.76–1.30)	-	-	-	
40–49 years	21 (1.69)	6 (1.51)	0.97 (0.46–2.03)	-	-	-	
Education level							
≤ primary school	445 (35.80)	117 (29.40)	1.00	1.00	1.00	1.00	
≥ secondary school	798 (64.20)	281 (70.60)	1.22 (1.00–1.49)	1.24 (1.02–1.51)	1.20 (0.98–1.46)	1.23 (1.04–1.47)	0.016
Parity							
Multiparous	607 (48.83)	160 (40.20)	1.00	-	-	-	
Primiparous	636 (51.17)	238 (59.80)	1.42 (0.66–3.03)	-	-	-	
Previous experience with breastfeeding							
Yes	589 (47.39)	155 (38.94)	1.00	-	-	-	
No	654 (52.61)	243 (61.06)	0.92 (0.43–1.98)	-	-	-	
Skin color							
Black/brown	1,013 (81.50)	322 (80.90)	1.00	-	-	-	
White	230 (18.50)	76 (19.10)	0.97 (0.77–1.22)	-	-	-	
Family income							
≥ 1 minimum salary	592 (47.63)	209 (52.51)	1.00	1.00	1.00	1.00	
< 1 minimum salary	651 (52.37)	189 (47.49)	0.82 (0.68–0.99)	0.95 (0.77–1.16)	0.97 (0.79–1.19)	0.95 (0.78–1.15)	0.631
Living with child’s father							
Yes	1,070 (86.08)	337 (84.67)	1.00	-	-	-	
No	173 (13.92)	61 (15.33)	1.13 (0.90–1.41)	-	-	-	
Number of prenatal visits							
≥ 6 visits	958 (77.07)	310 (77.89)	-	1.00		-	
< 6 visits	285 (22.93)	88 (22.11)	-	1.07 (0.87–1.32)		-	
Guidance on breastfeeding received during prenatal care							
Yes	332 (26.71)	91 (22.86)	-	1.00	-	-	
No	911 (73.29)	307 (77.14)	-	1.11 (0.91–1.36)	-	-	
Type of prenatal care							
Public/publicly funded	841 (67.66)	243 (61.06)	-	1.00	1.00	1.00	
Private	402 (32.34)	155 (38.94)	-	1.34 (1.07–1.69)	1.15 (0.82–1.60)	1.03 (0.82–1.30)	0.760
Type of delivery							
Vaginal	684 (55.03)	184 (46.23)	-	-	1.00	1.00	
Cesarean section	559 (44.97)	214 (53.77)	-	-	1.31 (1.07–1.61)	1.33 (1.13–1.57)	0.001
Type of hospital							
Public/publicly funded	920 (74.01)	271 (68.09)	-	-	1.00	-	-
Private	323 (25.99)	127 (31.91)	-	-	0.92 (0.63–1.33)	-	-
Delivery at Baby-Friendly Hospital Initiative-accredited facility							
Yes	312 (25.10)	66 (16.58)	-	-	1.00	1.00	
No	931 (74.90)	332 (83.42)	-	-	1.48 (1.09–2.00)	1.51 (1.15–1.99)	0.003
Breastfeeding within the first hour after delivery							
Yes	584 (46.98)	177 (44.47)	-	-	1.00	-	
No	659 (53.02)	221 (55.53)	-	-	1.06 (0.88–1.27)	-	
Birth weight							
≥ 2,500 g	1,185 (95.33)	381 (95.73)	-	-	1.00	-	
< 2,500 g	58 (4.67)	17 (4.27)	-	-	0.92 (0.61–1.40)	-	
Mother gets help with household chores							
Yes	940 (75.62)	304 (76.38)	-	-	-	1.00	
No	303 (24.38)	94 (23.62)	-	-	-	1.11 (0.92–1.34)	0.258
Father helps take care of the child							
Yes	1,047 (84.23)	326 (81.91)	-	-	-	1.00	
No	196 (15.77)	72 (18.09)	-	-	-	1.15 (0.94–1.42)	0.168
Type of nipple							
Normal	1,178 (94.77)	371 (93.22)	-	-	-	1.00	
Flat/inverted/pseudo-inverted	65 (5.23)	27 (6.78)	-	-	-	1.20 (0.90–1.61)	0.205
Fixed schedule to breastfeed at 30 days							
No	1,191 (95.82)	376 (94.47)	-	-	-	1.00	
Yes	52 (4.18)	22 (5.53)	-	-	-	1.09 (0.77–1.54)	0.619
Mother knows how to express excess milk at 30 days							
Yes	885 (71.20)	288 (72.36)	-	-	-	1.00	
No	358 (28.80)	110 (27.64)	-	-	-	1.02 (0.86–1.21)	0.752
Breastfeeding technique							
Proper	1,147 (92.28)	314 (78.89)	-	-	-	1.00	
Poor	96 (7.72)	84 (21.11)	-	-	-	3.18 (2.72–3.72)	0.000
Breast engorgement							
No	830 (66.77)	201 (50.50)	-	-	-	1.00	
Yes	413 (33.23)	197 (49.50)	-	-	-	1.70 (1.46–1.99)	0.000
Child’s sex							
Female	578 (46.50)	194 (48.74)	-	-	-	1.00	
Male	665 (53.50)	204 (51.26)	-	-	-	0.87 (0.75–1.01)	0.088
Use of a pacifier at 30 days							
No	734 (59.05)	219 (55.03)	-	-	-	1.00	
Yes	509 (40.95)	179 (44.97)	-	-	-	0.98 (0.84–1.16)	0.898
Use of a feeding bottle at 30 days							
No	1,010 (80.26)	297 (74.62)	-	-	-	1.00	
Yes	233 (18.74)	101 (25.38)	-	-	-	1.29 (1.06–1.55)	0.008

95 % CI 95 % confidence interval, *PR* prevalence ratio, ^a^Related to fourth-stage analysis

## Discussion

### Prevalence of cracked nipples in the first month postpartum

Even though cracked nipples are a long-known problem, and despite major advances in scientific and technological knowledge, the incidence of this condition continues to be extremely high. In the present study, 32 % of the women assessed presented cracked nipples in the first month postpartum. High rates have also been reported by other authors, e.g., 47 % of nipple trauma in the first week in one study conducted in southern Brazil [1], and 44 % at 30 days postpartum in another study [2]. The higher rates found in those studies can probably be explained by the fact that all types of nipple trauma were considered in that sample, vs. only cracked nipples in ours. Also, differences in the incidence of nipple pain/trauma across studies may be explained, at least in part, by the use of different definitions of nipple trauma and data collection methods. Some studies consider only lesions of continuity of the skin in the nipple-areolar region [8, 19], whereas others include any ulcer or skin abnormalities (cracks, excoriations, erosion, ecchymosis, spots, blisters) [2, 5], and yet others consider the mere presence of nipple pain or discomfort while breastfeeding [13, 20]. In the present study, only the presence of cracked nipples was taken into consideration, associated or not with pain, based on the mothers’ report.

### Factors associated with cracked nipples in the first month postpartum

An interesting finding of the present study was the fact that delivery and postnatal characteristics were more strongly associated with the occurrence of cracked nipples than individual and prenatal characteristics.

The variable most strongly associated with cracked nipples in the first month postpartum was poor breastfeeding technique. Women using a poor breastfeeding technique (according to the criteria here adopted) showed a three-fold higher chance of presenting cracked nipples in the first month postpartum. Even though the design of the present study does not allow to state that a causal relationship exists between poor breastfeeding technique and nipple trauma, there is consensus that a proper breastfeeding technique is the most effective preventive strategy and treatment for this outcome [7–9, 12, 13, 20]. The few studies that have investigated specific determinants of cracked nipples have also pointed to an association between nipple trauma and poor breastfeeding technique [5, 6].

Two other postnatal variables were associated with cracked nipples: breast engorgement and use of a feeding bottle. The association between cracked nipples and breast engorgement has been previously reported [8, 9]. Breast engorgement changes the anatomy of the nipple region, hindering proper latch-on due to swelling, turgidity, little flexibility, and flat nipples [8]. From a different standpoint, nipple trauma can also cause or worsen breast engorgement, as the pain experienced by the mother may interfere with the frequency and duration of feeds [8].

The use of a feeding bottle, in turn, may be associated with cracked nipples as a consequence of the different sucking techniques required for the bottle and the breast. When fed with a bottle, the child positions her tongue so as to control the flow of milk; use of the same movement on the breast may result in nipple trauma. This association has been demonstrated by Centouri et al. [7] in Italy and by França et al. [1] in Brazil. Notwithstanding, once again, the reverse causality in this relationship should not be disregarded: it is possible that the pain caused by nipple trauma has led the mothers to offer the bottle to their children [1].

Two variables related to delivery care were associated with presence of cracked nipples: cesarean section and delivery at a hospital not accredited by the Baby-Friendly Hospital Initiative. The association between a cesarean section and cracked nipples has been confirmed in some studies [21] and refused in others [10, 19]. It is possible that the pain caused by the surgical incision may affect positioning of the mother-child dyad during breastfeeding. Nevertheless, it has been demonstrated that women who had a cesarean section show better breastfeeding performance when they are assisted in controlling the pain and correcting the child’s position while breastfeeding [22]. The use of anesthetics during the surgical procedure may also interfere with the child’s ability to suck [10].

Another important finding of our sample that has not been explored in the few previous studies focusing on this topic is the fact that birth at a maternity ward not accredited by the Baby-Friendly Hospital Initiative was a factor associated with cracked nipples. This finding is not surprising, as the routines of accredited hospitals include preventive actions against nipple trauma, e.g., providing guidance on proper breastfeeding techniques, on how to express milk in case of breast engorgement, in addition to the recommendation to avoid the use of a pacifier/bottle [23].

The only individual variable significantly associated with cracked nipples in our sample was higher maternal education level. In Brazil, women with higher education levels have been shown to maintain exclusive breastfeeding for longer [24, 25] – a finding that probably reflects greater awareness of the importance of breastfeeding and a better understanding of the guidance received from health professionals. As a result, we expected that higher maternal education level would protect against cracked nipples, when in fact the opposite was observed. This finding could be related to the fact that the occurrence of cracked nipples was self-reported by the mothers, i.e., the information could be biased [26]. Further studies are necessary to confirm and better understand this association, investigating aspects not covered in the present study, e.g., nipple pigmentation and mother’s perceptions and appreciation regarding cracked nipples.

### Methodological considerations

One methodological strength of the study is the statistical analysis, in which variables were organized into different levels, allowing to demonstrate that the outcome was more strongly influenced by proximal characteristics. Also, all the professionals involved in data collection were specifically trained for the task and were able to assess and classify the breastfeeding technique according to the criteria established. Conversely, the outcome of interest, cracked nipples, was self-reported by mothers, which may have led to a measurement bias. In our opinion, however, this limitation does not affect the relevance of the study, as we strongly believe that the perception and report of mothers with regard to the presence of cracked nipples is reliable. Moreover, if some women in our sample had cracked nipples but chose not to report the condition, it is likely that the condition had a lower impact on their breastfeeding performance.

## Conclusions

In sum, this study adds to the existing body of knowledge on the factors associated with cracked nipples in the first month postpartum and can help design prevention strategies. According to the present findings, to decrease the prevalence of cracked nipples, it is important to support vaginal delivery and improve the care provided to women who undergo a cesarean section, to incorporate the routines advocated by the Baby-Friendly Hospital Initiative at maternity wards, to guarantee that mothers leave the maternity ward well informed of the proper breastfeeding technique and of how to avoid breast engorgement, and to discourage the use of feeding bottles, regardless of the mother’s education level. Finally, it is extremely important that mothers receive appropriate support from trained health professionals with breastfeeding expertise, both in the immediate postpartum period and after discharge.

## Abbreviations

95 % CI, 95 % confidence interval; PR, prevalence ratio; SPSS, Statistical Package for the Social Sciences
